# Conflicting health information increases fear of disease progression in HPV-infected individuals: disease uncertainty as mediator and tolerance of uncertainty as buffer

**DOI:** 10.3389/fpubh.2025.1532592

**Published:** 2025-02-12

**Authors:** Li Huai, Sihua Liao, Xiaokang Lyu, Tingting Yang, Chunye Fu

**Affiliations:** ^1^Department of Gynecology, The Second Affiliated Hospital of Wenzhou Medical University, Wenzhou, Zhejiang, China; ^2^Department of Social Psychology, School of Sociology, Nankai University, Tianjin, China

**Keywords:** HPV infection, conflicting health information, fear of progression, disease uncertainty, tolerance of uncertainty

## Abstract

**Background:**

Human papillomavirus (HPV) infection is highly prevalent, and infected individuals frequently encounter HPV-specific conflicting health information about their condition. Although this phenomenon is well documented, the mechanisms by which such information influences fear of progression (FoP) and potential interventions remain unexplored.

**Methods:**

This research was conducted in two phases. Phase 1 (*n* = 218) developed and validated the HPV-Specific Conflicting Health Information Scale. Phase 2 (*n* = 501) examined disease uncertainty as a mediator and tolerance of uncertainty as a moderator among HPV-positive female patients.

**Results:**

The HPV-Specific Conflicting Health Information Scale demonstrated a two-dimensional structure representing objective experiences and subjective perceptions of HPV-specific conflicting health information. Phase 2 revealed that HPV-specific conflicting health information indirectly influenced Fear of Progression via disease uncertainty, with this relationship moderated by individuals’ tolerance of uncertainty.

**Conclusion:**

This study established the first validated measure of HPV-specific conflicting health information. The findings indicate that enhancing tolerance of uncertainty may serve as an effective complement to reducing conflicting information exposure in addressing HPV-related psychological distress.

## 1 Introduction

HPV infection remains a pressing public health concern, with HPV screening being identified by the World Health Organization as one of the key strategies for eliminating cervical cancer. Human papillomavirus (HPV), a double-stranded DNA virus, is responsible for almost all cases of cervical cancer worldwide (1, 2). Statistics indicate that 80% of men and 90% of women will infect HPV at some point in their lives (3). In 2020, an estimated 604,127 cervical cancer cases and 341,831 deaths were reported globally (4), with new cases increasing to approximately 660,000 in 2022 (5).

In 2020, the World Health Organization launched a global initiative to eliminate cervical cancer through a comprehensive approach including HPV vaccination, screening, and treatment of diagnosed cervical diseases (6). In response to this call, many countries have developed and implemented cervical cancer screening guidelines and practices, with HPV testing playing a crucial role in these initiatives (2, 7).

HPV screening, despite its widespread clinical application, faces significant challenges in diagnostic specificity and procedural complexity. These challenges manifest in three key areas. First, while HPV testing demonstrates high sensitivity in viral detection, it has limited specificity in discriminating between high-risk and low-risk genotypes (8), potentially resulting in overdiagnosis and subsequent overtreatment (9). Second, screening protocols lack international standardization, with substantial variations across countries in recommended screening ages, intervals, and methodologies (2). Third, continuous advancements in HPV detection technologies necessitate frequent protocol updates to address issues of testing optimization and diagnostic accuracy (10).

Individuals with HPV frequently encounter conflicting health information, both passively and actively. A typical example occurs when HPV-related information on social media platforms contradicts medical advice: physicians may recommend monitoring certain HPV infections without intervention, while social media advocates immediate treatment. Carpenter and Han (11) systematically conceptualize conflicting health information as two or more logically inconsistent health-related propositions and identify four fundamental dimensions: substantive issues, source diversity, evidence heterogeneity, and temporal inconsistency. In the HPV context, these dimensions are reflected in various ways: substantive issues manifest as differences in prevention, diagnosis, and treatment protocols; source diversity appears as contradictions within or between information sources; evidence heterogeneity emerges from inconsistent clinical research findings; and temporal inconsistency arises from dynamic guideline updates.

Conflicting health information involves not only clear contradictions in the content itself but also individuals’ subjective perceptions of these conflicts. Zimbres et al. (12) created a model that differentiates between objective conflicting information that one encounters and the subjective experience of perceiving such conflicts. Their model illustrates that simply being exposed to conflicting information does not automatically lead to feelings of conflict; these objective and subjective experiences can produce different outcomes. Therefore, in this study we define conflicting health information in relation to HPV as including both the actual experience of encountering conflicting HPV-related health information and the psychological distress that may arise from perceiving these conflicts. This dual-dimensional conceptualization acknowledges that even when individuals come across the same conflicting health information, their subjective perceptions and psychological reactions to these contradictions can differ significantly. Despite the theoretical importance of these distinctions, there are currently no validated tools available to systematically assess individuals’ experiences with HPV-related conflicting information.

Exposure to conflicting health information generates multiple adverse effects. At the psychological level, such information elicits negative emotional responses including confusion, frustration, and anger (13), while simultaneously disrupting cognitive attention mechanisms crucial for accurate information processing (14). These effects demonstrate a spillover phenomenon, where cognitive resistance extends beyond the conflicting information to affect the processing of other well-evidenced health information (13).

The adverse effects on health-related behaviors are particularly pronounced. Increased exposure to conflicting medication information negatively correlates with medication adherence (15, 16). Similarly, contradictory expert opinions diminish trust in expertise and reduce compliance with professional recommendations (17). Studies indicate that such exposure decreases health information seeking and sharing behaviors (18) and undermines self-efficacy in cancer prevention practices (19). In the HPV context, exposure to contradictory information impairs cognitive processing of vaccine information and elevates confusion, subsequently reducing vaccination intentions through increased decision conflict (20). Parallel findings in breast cancer screening research demonstrate that conflicting media information heightens confusion and negative attitudes toward mammography (21).

Fear of progression (FoP) represents the most characteristic psychological state among HPV-infected individuals. Fear of progression was initially conceptualized to describe cancer patients’ concerns about disease recurrence, progression, and metastasis (22). Unlike general health anxiety, FoP is a multidimensional construct encompassing emotional responses (such as worry and anxiety), partner and family relationships (impacts on family members), occupational effects (changes in work capability), and loss of autonomy.

Although HPV infection does not inevitably lead to cancer, the possibility of progression makes FoP one of the most common psychological burdens for HPV-positive individuals (23). Research evidence demonstrates the prevalence of this multidimensional fear among HPV-infected individuals. A German study of patients with HPV-related premalignant genital lesions found that 22% of women “often” or “very often” experienced anxiety about disease progression, while 47% frequently experienced physical symptoms of fear. Notably, these patients showed no significant differences from cancer patients in partner-related or family-related concerns (24). Through interviews with women with abnormal Pap smears (a screening result highly correlated with HPV infection), Rask et al. (25) found that these women tended to interpret abnormal results as indicators of cancer, leading to anxiety. Ciavattini et al. (26) online survey similarly found that anxiety, worry, and fear were the most commonly reported emotions among women with HPV-positive or abnormal Pap smear results. In China, Li et al. (27) identified through interviews that HPV-positive women’s fear of progression primarily manifested as emotional responses, concerns about physiological health, and worries about family and social functioning.

Existing evidence suggests that conflicting health information may exacerbate fear of progression among HPV-infected individuals. Exposure to conflicting health information triggers negative emotional responses, including confusion, frustration, and worry (13). Han et al. (28) found that exposure to inconsistent health information increases cancer risk perception and related concerns, even among individuals with no cancer history. This impact may be particularly threatening for HPV-infected individuals for two reasons: first, HPV infection has potential links to cancer development (29), and second, research has demonstrated HPV-infected individuals commonly experience significant fear of progression (23).

Disease uncertainty may represent a pathway linking conflicting health information to fear of progression. The Uncertainty in Illness Theory identifies three key factors that influence individuals’ assessment of uncertainty: lack of credible authority, event uncertainty, and absence of symptom patterns (30). These factors directly correspond to characteristics of conflicting health information: content conflicts undermine authority, evidence heterogeneity increases uncertainty, and temporal inconsistency prevents pattern recognition.

In the context of HPV infection, conflicting health information serves as uncertainty cues that hinder the formation of cognitive schemas about the disease process. This difficulty in constructing coherent understanding leads to uncertainty about disease unpredictability, which may trigger systematic concerns about disease progression, including worries about physical health, career impact, and family functioning (31).

Tolerance of uncertainty may serve as a protective factor in the process through which illness uncertainty influences fear of progression. Tolerance of Uncertainty refers to an individual’s capacity to endure uncertain situations, manifested in individual differences across cognitive, emotional, and behavioral responses (32, 33). These individual differences can influence how people evaluate and cope with uncertainty (34). In health contexts, lower tolerance of uncertainty significantly correlates with higher levels of anxiety and depression, as demonstrated during the high-uncertainty environment of the COVID-19 pandemic (35).

Research in cancer contexts has also examined the role of uncertainty tolerance in the development of fear of progression. Waroquier et al. (36) found a significant correlation between cancer patients’ intolerance of uncertainty and their fear of progression. From a stress response perspective, Shen et al. (37) discovered that illness uncertainty might increase fear of progression by reducing individuals’ tolerance of uncertainty. The present study proposes that tolerance of uncertainty, as a relatively stable individual trait, may influence how individuals evaluate and cope with illness uncertainty, thereby moderating its impact on fear of progression.

Complex relationships exist among HPV-related conflicting health information, illness uncertainty, and fear of progression. However, current research has two major limitations. First, despite HPV-infected individuals’ extensive exposure to conflicting health information, there is no dedicated scale for assessing such experiences and perceptions. Second, while research has demonstrated that conflicting health information triggers negative emotional responses, and the Uncertainty in Illness Theory provides a theoretical framework for understanding this process, a systematic examination of the mechanisms underlying fear of progression in HPV-infected individuals is lacking. Notably, the role of tolerance of uncertainty as a potential protective factor in this process remains unexplored.

Therefore, this study will proceed in two phases: initially developing and validating an HPV-Specific Conflicting Health Information Scale, followed by investigating the mechanism through which conflicting health information influences fear of progression via illness uncertainty, and examining the moderating role of tolerance of uncertainty. This research will not only deepen understanding of the psychological state of HPV-infected individuals but also provide a foundation for developing targeted psychological interventions.

**Figure 1:**
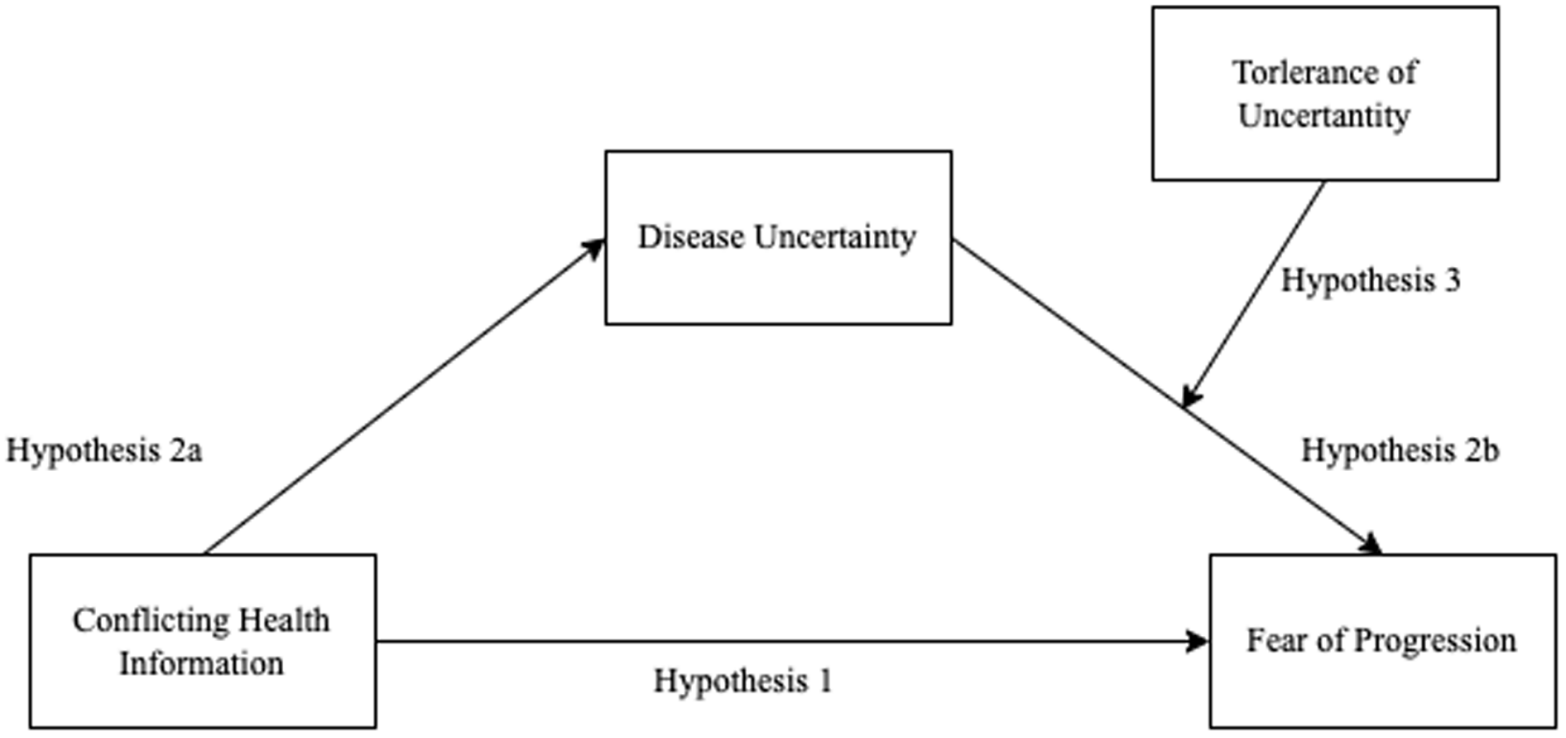
Conceptual model of the relationships between conflicting health information, disease uncertainty, tolerance of uncertainty, and fear of progression.

To address these research questions, this study proposes the following hypotheses (Figure 1):

Hypothesis 1: Conflicting health information positively correlates with fear of progression in HPV-positive individuals.

Hypothesis 2: Disease uncertainty mediates the relationship between conflicting health information and fear of progression.

Specifically:

Hypothesis 2a: Conflicting health information positively predicts disease uncertainty.

Hypothesis 2b: Disease uncertainty positively predicts fear of progression.

Hypothesis 3: Tolerance of uncertainty moderates the effect of disease uncertainty on fear of progression, such that higher tolerance of uncertainty weakens the positive impact of disease uncertainty on fear of progression.

## 2 Materials and methods

### 2.1 Procedures

The research was conducted in two main phases. Phase 1 comprised two steps. Step 1 involved the development of an initial pool of seven items based on common HPV-related patient inquiries (38). Semi-structured interviews with 13 HPV-positive female patients were conducted to refine these items and explore potential new ones. Two rounds of expert consultation further refined the scale. A draft scale was then generated using a dual-response format for each item, based on interview findings and expert feedback. Step 2 of Phase 1 focused on psychometric validation. Between September and October 2023, 218 HPV-positive female patients completed the draft scale. Item analysis, exploratory factor analysis (EFA), and confirmatory factor analysis (CFA) were performed to establish the scale’s final structure. Test–retest reliability was assessed with 42 participants over a 1–2-week interval.

**Figure 2:**
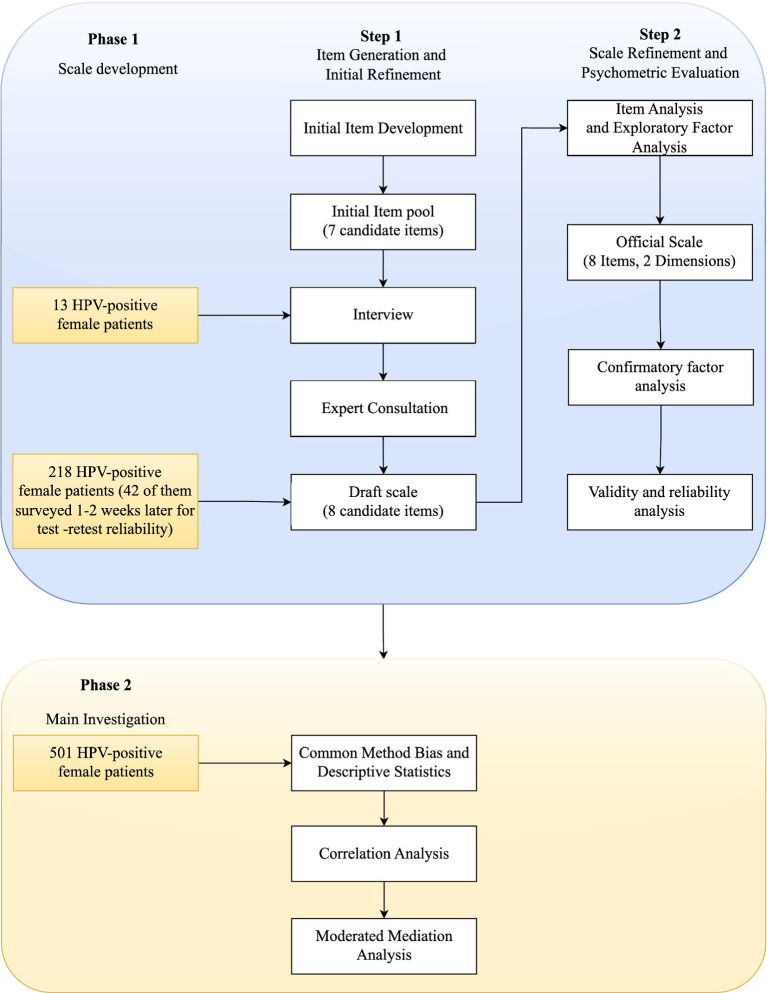
Flowchart of HPV-Specific Conflicting Health Information Scale development and study methodology.

Phase 2 involved 501 HPV-positive female patients to examine relationships among HPV-related conflicting health information, fear of disease progression, disease uncertainty, and tolerance of uncertainty using the validated scale. The study was approved by institutional board of Nankai University. All participants provided informed consent before participation. The study flowchart is presented in Figure 2.

### 2.2 Phase 1: Scale development

#### 2.2.1 Step 1: Item generation and initial refinement

##### 2.2.1.1 Initial item development

The development of the HPV-Specific Conflicting Health Information Scale was necessitated by the absence of existing measures specifically designed for this purpose. Adapting Carpenter et al. (39) method for assessing conflicting drug information, an initial pool of seven items was developed based on the most frequently encountered uncertain and conflicting HPV-related questions from patients in clinical practice (see Table 1).

**Table 1:** Proposed items for HPV-Specific Conflicting Health Information Scale.

Item number	Item content
(1)	Some sources indicate a low probability of cervical cancer from HPV infection, yet I’ve encountered case reports of HPV-induced cervical cancer.
(2)	While some information suggests a low likelihood of HPV infection leading to cervical cancer, I’ve also encountered reports of severe consequences following HPV infection.
(3)	Information promoting HPV vaccination often emphasizes the severe consequences of infection, whereas some HPV educational materials state that most infections resolve spontaneously.
(4)	When researching the relationship between HPV infection and cervical cancer, I’ve encountered inconsistent medical research findings.
(5)	Some sources suggest that most HPV infections can be resolved by the immune system without undue concern, while others emphasize the importance of treatment following infection.
(6)	Certain sources advise against frequent examinations post-HPV infection, while others recommend regular check-ups to prevent disease progression.
(7)	Some information advocates for pharmacological interventions post-HPV infection, while other sources promote surgical treatments as more effective.

Items were originally developed in Chinese and are presented here in English translation. The scale was designed for use with Chinese-speaking populations.

Informed by Zimbres et al. (12) research on Contradictory Health Information Processing, which highlighted the distinction between objective contradictions in health information and subjective perceptions, a dual-response format was implemented. Each of the seven initial items was rated on two five-point Likert scales, measuring the degree of congruence with personal experience (from 1 “completely incongruent” to 5 “completely congruent”) and the level of distress caused by such conflicting health information (from 1 “no distress” to 5 “extreme distress”). This dual-response format enabled the assessment of both objective experiences and subjective perceptions of HPV-related conflicting health information.

Table 2 reveals that among intra-source comparisons, patients encountered conflicting health information most frequently in social media (*M* = 3.54, *SD* = 1.32), which also caused the highest level of distress (*M* = 3.00, *SD* = 1.23). For inter-source comparisons, patients reported the highest frequency of information conflicts between social media and doctors’ advice (*M* = 3.15, *SD* = 0.43), accompanied by the highest level of distress (*M* = 2.69, *SD* = 0.49). These findings indicate that psychological distress resulting from conflicting health information exists objectively and impacts patients’ subjective perceptions. These findings underscore the prevalence of conflicting health information, particularly involving social media, and its potential to cause distress among HPV-positive patients.

**Table 2:** Patients’ experiences and perceptions of conflicting health information from same and different sources.

Information source	Measure	*M*	*SD*
Within single information source			
Social media	Inconsistency	3.54	1.32
	Distress level	3.00	1.23
Medical literature	Inconsistency	1.85	0.38
	Distress level	2.69	0.40
Different doctors	Inconsistency	2.69	0.41
	Distress level	2.62	0.40
Between different information sources			
Social media source vs. medical literature source	Inconsistency	2.77	0.51
	Distress level	2.54	0.34
Social media source vs. doctor’s information source	Inconsistency	3.15	0.43
	Distress level	2.69	0.49
Medical literature source vs. doctor’s information source	Inconsistency	1.62	0.30
	Distress level	2.46	0.41

##### 2.2.1.2 Interview

To refine the initial scale items, semi-structured interviews were conducted with 13 HPV-positive female patients (age range: 26–47 years). Participants were recruited through purposive sampling from September 20 to September 30, 2023, at the Minimally Invasive Gynecology Center of a Class-A Tertiary Hospital in Wenzhou, Zhejiang Province, China.

The interview protocol focused on two questions: “What are your usual sources of information about HPV?” and “What sources of inconsistent information are you aware of, including inconsistencies within and between different information sources?”

The interviews confirmed that participants’ information sources were consistent with those identified in our initial scale development: social media, doctors’ advice, and medical literature, with no additional sources emerging. Although some participants shared experiences of encountering conflicting information about HPV infection outcomes, these narratives fell within the scope of Item 2 in our existing scale. Therefore, no new items were identified during the interview process.

##### 2.2.1.3 Expert consultation

The initial scale underwent two rounds of expert consultation for refinement. The expert panel consisted of 11 female professionals in the first round (response rate: 90.90%) and 10 in the second round (response rate: 100%). These experts (*M*_age_ = 40.80 years, *SD* = 4.66; *M*_work experience_ = 15.70 years, *SD* = 7.44) included three social psychology experts, four clinical doctors specializing in cervical diseases, one head nurse in gynecological clinical care, and two directors in relevant nursing education and research. All experts were female.

In the first round of expert consultation, two social psychology experts suggested that items 1 and 2 were measuring the same content and could be merged into one item. One nursing education expert suggested rewording item 2 to begin with “Some information suggests HPV infection is not serious” and modifying item 7 (item 6 in final scale) to contrast “Only medication is needed” with “surgical treatment is superior to medication.” A clinical cervical disease expert suggested adding a new item (item 7) addressing conflicting information about HPV clearance: “Some sources suggests that HPV can be cleared after treatment, while others state that even after medication or surgical treatment, clearance is not guaranteed.” One clinical nursing expert recommended adding an item (item 8) about conflicts between HPV vaccination and infection: “Some information suggests that vaccination after HPV infection is of little significance, while other sources claim that HPV vaccination post-infection still provides protection.”

Based on expert feedback, the scale was refined by merging and modifying items 1 and 2 from the initial scale, adjusting items 6 and adding items 7 and 8.

In the second round of expert consultation, all experts endorsed our items and the two-dimensional classification of all items. They provided no additional suggestions. The consultation process demonstrated high authority (coefficients: 0.82 and 0.81 for rounds one and two, respectively) and significant coordination (degrees: 0.237 and 0.178, *p* < 0.05). All items met the screening criteria (average importance score ≥ 3.5, coefficient of variation <0.25).

The final product was an 8-item HPV-Specific Conflicting Health Information Scale (see Table 3). Each item comprises two sub-questions assessed on 5-point Likert scales (1 = “completely incongruent/no distress,” 5 = “completely congruent/high distress”).

**Table 3:** Final items of the HPV-Specific Conflicting Health Information Scale.

Number	Item content
(1)	Some information suggests that HPV infection is not serious, but I have also seen case reports of severe consequences, including cervical cancer, following HPV infection.
(2)	Some information promoting HPV vaccination emphasizes the severe consequences of infection, while other HPV educational materials state that most HPV infections resolve on their own.
(3)	When researching the relationship between HPV infection and cervical cancer, I have encountered inconsistent medical research findings.
(4)	Some sources suggest that most HPV infections can be resolved by the immune system without undue concern, while others emphasize the importance of treatment following infection.
(5)	Certain sources advise against frequent examinations post-HPV infection, while others recommend regular check-ups to prevent disease progression.
(6)	Some information suggests that HPV infection only requires medication, while other sources state that surgical treatment is superior to medication.
(7)	Some sources suggests that HPV can be cleared after treatment, while others state that even after medication or surgical treatment, clearance is not guaranteed.
(8)	Some information suggests that vaccination after HPV infection is of little significance, while other sources claim that HPV vaccination post-infection still provides protection.

#### 2.2.2 Step 2: Scale refinement and psychometric evaluation

##### 2.2.2.1 Participants

Using convenience sampling, a sample (*N* = 218, *M*_age_ = 32.33 years) was recruited, consisting of individuals with high-risk HPV positivity or persistent HPV infection, with or without TCT abnormalities.

##### 2.2.2.2 Statistical analysis

Item analysis and scale validation involved a multi-step process. First, item quality was evaluated using three methods: (1) critical ratio method, where participants were divided into high-score (top 27%) and low-score (bottom 27%) groups, with item discrimination assessed using Student’s t-test; (2) item-total correlations, examining the relationship between each item and the total score; and (3) factor loading analysis. All items met the retention criteria (critical ratio > 3.0, item-total correlations > 0.3, and factor loadings > 0.4).

Exploratory factor analysis (EFA) was then performed using SPSS to examine the scale’s structure. Data suitability was assessed through the KMO test (criterion: >0.6) and Bartlett’s test of sphericity (*p* < 0.05). Principal component analysis with varimax rotation extracted factors with eigenvalues >1. The cumulative variance contribution exceeded 40%, with all items showing loadings above 0.4 and no significant cross-loadings.

Confirmatory factor analysis (CFA) validated the factor structure derived from EFA, evaluating model fit through *χ*^2^/df (<3), CFI and TLI (>0.9), RMSEA and SRMR (<0.08). Scale structure was further validated by analyzing inter-dimensional and scale-dimension correlations.

Reliability was assessed via Cronbach’s *α* (>0.7) for internal consistency and intraclass correlation coefficient (>0.75) for test–retest reliability, with a 1–2 weeks interval between administrations for a subset of 42 participants.

### 2.3 Phase 2 main investigation

This phase aimed to examine the relationship between conflicting health information and fear of disease progression in HPV-positive individuals through a survey using the HPV-Specific Conflicting Health Information Scale developed in the previous phase.

#### 2.3.1 Participants

A convenience sampling method was employed to select participants from the colposcopy diagnosis and treatment center of a Class-A Tertiary Hospital in Wenzhou. Eligible participants were 18 years or older (*M*_age_ = 38.89 ± 9.84 years), with high-risk HPV positivity or persistent HPV infection, with or without TCT abnormalities. Individuals with cervical cancer or precancerous lesions, as well as those lacking self-behavioral capacity or with mental disorders, were excluded from the study. A total of 501 HPV-positive individuals who agreed to participate were surveyed using a combination of online questionnaires (via Wenjuanxing platform) and paper questionnaires (Table 4).

**Table 4:** Participants’ demographic information.

Characteristic	Category	*n*	%
Marital status	Single	49	9.8
	Married	452	90.2
Number of children	None	65	13.0
	1	165	32.9
	2	226	45.1
	3 or more	45	9.0
Contraception method	None	120	24.0
	Condom	170	33.9
	Other	211	42.1
Education level	Primary school or below	80	16.0
	Middle school	166	33.1
	High school/Vocational school	107	21.4
	College or above	148	29.5
HPV type	High-risk	449	89.6
	Low-risk	52	10.4
HPV vaccination	Yes	128	25.5
	No	373	74.5
TCT test	Normal	253	50.5
	Abnormal	248	49.5
Duration of infection	Less than 3 months	200	39.9
	3–12 months	84	16.8
	More than 12 months	217	43.3

#### 2.3.2 Materials

This study utilized four scales to assess the key variables:

**HPV-Specific Conflicting Health Information Scale:** Developed in the initial phase of this study, this 8-item scale measures two dimensions: objective conflict experience and subjective conflict perception. In the current study, the scale demonstrated high internal consistency (total scale: *α* = 0.90; objective conflict experience: *α* = 0.89; subjective conflict perception: *α* = 0.88).

**Mishel Uncertainty in Illness Scale (MUIS):** This 20-item scale, originally developed by Mishel (40) and adapted for Chinese populations by Ye et al. (41), assesses illness-related uncertainty. Responses are recorded on a 5-point Likert scale, with five items reverse-scored. Total scores range from 20 to 100, with higher scores indicating greater disease uncertainty. The scale showed excellent internal consistency in this study (*α* = 0.91).

**Tolerance of Uncertainty Scale-Short Form (IUS-12):** Developed by Carleton et al. (42) and validated in Chinese by Zhang et al. (43), this 12-item scale measures individuals’ responses to uncertainty using a 5-point Likert scale. Higher scores reflect lower tolerance for uncertainty. The scale demonstrated high reliability in the current study (*α* = 0.93).

**Fear of Disease Progression Scale for HPV-Positive Women:** This 15-item scale, developed and validated by Li et al. (27) based on Mishel’s Uncertainty in Illness Theory, assesses three dimensions: emotional response, physiological health, and family and social function. Responses are recorded on a 5-point Likert scale, with higher scores indicating greater fear of disease progression. The scale showed strong internal consistency in this study (*α* = 0.90).

#### 2.3.3 Statistical analysis

In Phase 2, the main investigation analysis comprised several steps. First, descriptive statistics were calculated to provide basic characteristics of all variables, including means, standard deviations, and ranges. Next, Pearson correlation coefficients were computed to preliminarily examine bivariate relationships among study variables. Finally, to test the proposed theoretical model, a moderated mediation analysis was conducted using PROCESS macro 3.3 [Model 14; (44)] in SPSS 26.0. This analysis examined the relationships among conflicting health information (independent variable), disease uncertainty (mediator), tolerance of uncertainty (moderator), and fear of disease progression (dependent variable). Confidence intervals (95%) that did not include zero indicated significant effects. For all analyses, *p* < 0.05 was considered statistically significant.

## 3 Results

### 3.1 Results of scale development

#### 3.1.1 Item analysis results

Table 5 presents the item analysis results. All items demonstrated critical ratio values exceeding 3, item-total correlations greater than 0.4, and factor loadings above 0.45. These findings indicate that all items met the retention criteria for the scale.

**Table 5:** Summary of item analysis.

Item	Critical Ratio (CR)	Correlation coefficient	Factor loading	Decision
X1.1	8.69**	0.61	0.73	Keep
X1.2	8.12**	0.65	0.80	Keep
X2.1	12.39**	0.75	0.87	Keep
X2.2	10.40**	0.70	0.86	Keep
X3.1	12.51**	0.76	0.87	Keep
X3.2	7.74**	0.64	0.54	Keep
X4.1	12.96**	0.78	0.95	Keep
X4.2	9.24**	0.67	0.75	Keep
X5.1	14.63**	0.80	0.96	Keep
X5.2	7.13**	0.55	0.55	Keep
X6.1	13.29**	0.55	0.95	Keep
X6.2	9.28**	0.78	0.60	Keep
X7.1	14.15**	0.63	0.97	Keep
X7.2	7.76**	0.58	0.54	Keep
X8.1	14.63**	0.81	0.90	Keep
X8.2	9.86**	0.61	0.78	Keep
Standard	>3	>0.4	>0.45	

#### 3.1.2 Validity analysis results

##### 3.1.2.1 Exploratory factor analysis (EFA)

EFA was conducted on the 16 items of the scale. The Kaiser–Meyer–Olkin (KMO) measure of sampling adequacy was 0.91, and Bartlett’s test of sphericity was significant (*χ*^2^ = 5,835.61, *p* < 0.001), indicating the data were suitable for factor analysis. Principal component analysis with varimax rotation extracted two factors with eigenvalues greater than 1, accounting for 74.16% of the total variance.

**Figure 3:**
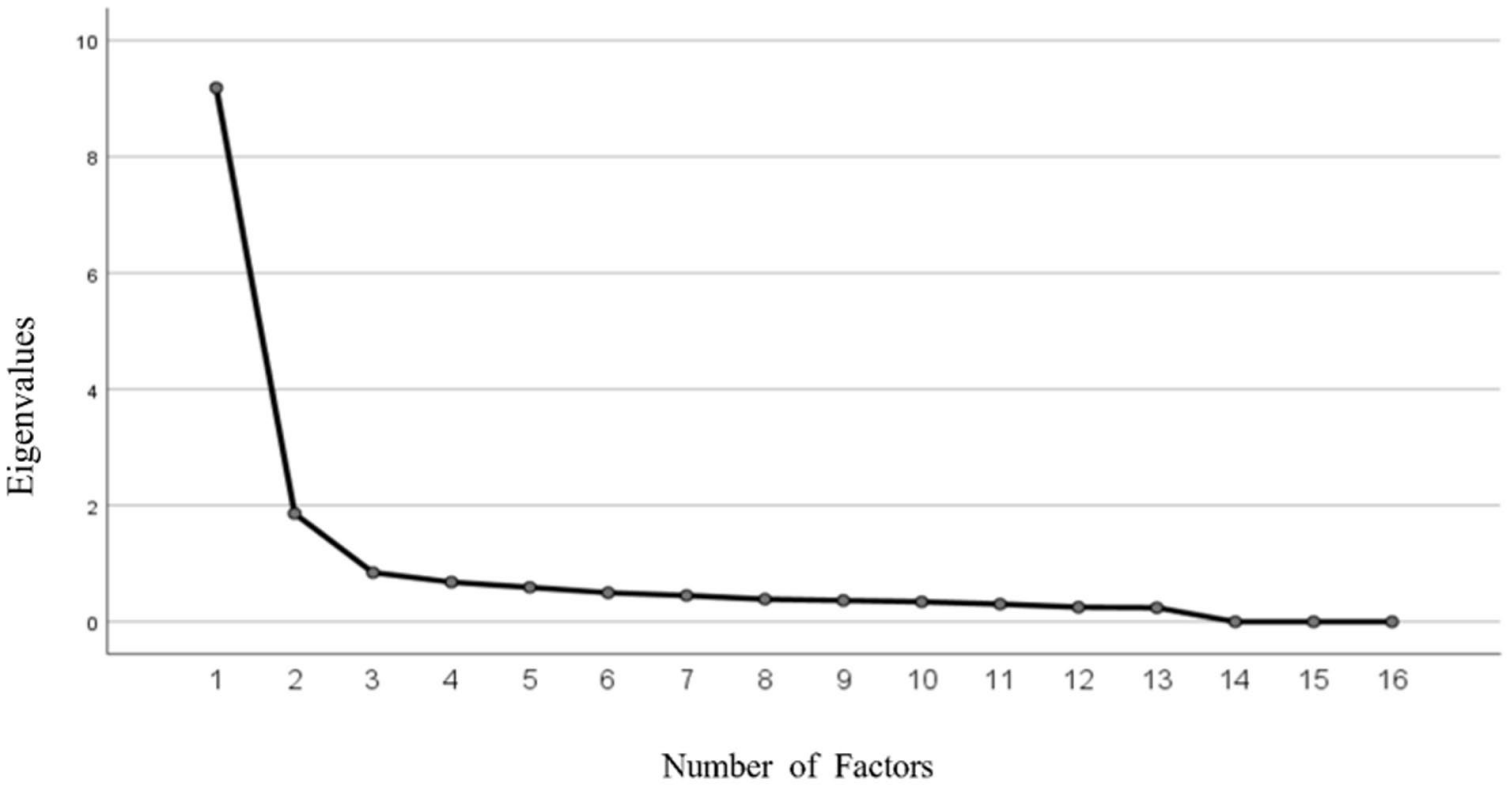
Scree plot of eigenvalues.

Based on the rotated factor matrix, the HPV-Specific Conflicting Health Information Scale was divided into two dimensions (see Table 6; Figure 3).

**Table 6:** Exploratory factor analysis results for the HPV-Specific Conflicting Health Information Scale.

Item	Factor 1	Factor 2
X5.1	0.85	0.19
X8.1	0.85	0.21
X7.1	0.87	0.20
X6.1	0.90	0.19
X4.1	0.90	0.19
X2.1	0.87	0.16
X3.1	0.86	0.10
X1.1	0.79	0.08
X2.2	0.10	0.87
X1.2	0.05	0.89
X8.2	0.10	0.88
X4.2	0.12	0.85
X6.2	0.03	0.76
X5.2	0.11	0.74
X7.2	0.10	0.73
X3.2	0.20	0.71
Eigenvalue	7.42	4.28
Explained variance	46.36%	26.79%

Factor 1, labeled “Objective Conflict Experience,” consisted of eight items (X1.1, X2.1, X3.1, X4.1, X5.1, X6.1, X7.1, X8.1) and explained 46.36% of the total variance. These items reflected the frequency of objectively experiencing HPV information conflict.

Factor 2, labeled “Subjective Conflict Perception,” comprised eight items (X1.2, X2.2, X3.2, X4.2, X5.2, X6.2, X7.2, X8.2) and accounted for 26.79% of the total variance. These items reflected the subjective perception of conflict after experiencing conflicting HPV information.

##### 3.1.2.2 Confirmatory factor analysis (CFA)

A CFA was conducted to assess the structural validity of the two-dimensional model. The results, presented in Table 7, indicate that all fit indices met the recommended criteria, suggesting good structural validity of the two-factor model.

**Table 7:** Results of confirmatory factor analysis for the HPV-Specific Conflicting Health Information Scale.

	*χ*^2^/df	CFI	TLI	RMSEA	SRMR
Model 1	2.29	0.98	0.96	0.02	0.03

##### 3.1.2.3 Interdimensional and scale-dimension correlations

Table 8 presents the results of interdimensional and scale-dimension correlations. The correlation coefficient between the two dimensions was 0.62. Correlations between each dimension and the total scale score were approximately 0.80. These findings suggest good internal structure of the scale.

**Table 8:** Correlation matrix of dimensions and total scale.

	Total score	Objective conflict experience	Subjective conflict perception
Total score	1		
Objective conflict experience	0.74**	1	
Subjective conflict perception	0.79**	0.62**	1

#### 3.1.3 Reliability analysis results

##### 3.1.3.1 Internal consistency

The overall Cronbach’s *α* coefficient for the scale was 0.90. The Cronbach’s *α* coefficients for the “Objective Conflict Experience” and “Subjective Conflict Perception” dimensions were 0.89 and 0.88, respectively.

##### 3.1.3.2 Test–retest reliability

A subset of 42 participants completed a follow-up assessment after 2 weeks. The test–retest reliability coefficient was 0.89, indicating good temporal stability of the scale.

The final scale comprises eight items, each with two questions: (1) Degree of alignment with actual experience, rated on a 5-point scale from “Completely Disagree” to “Completely Agree”; and (2) Level of distress caused by experiencing or hypothetically experiencing such inconsistent information, rated on a 5-point scale from “None” to “Very High.”

The psychometric properties of the scale meet the standards for psychological measurement, supporting its use as a tool for measuring women’s experiences and perceptions of HPV-related conflicting health information.

### 3.2 Main investigation results

#### 3.2.1 Common method bias and descriptive statistics

To address potential common method bias, this study employed both procedural and statistical approaches. Procedurally, participants were informed about the study’s purpose and anonymity, and data collection timing was varied. Statistically, Harman’s single-factor test was conducted. The results revealed nine factors with eigenvalues greater than 1, with the first factor accounting for 34.83% of the total variance. This indicates that common method bias was not a significant concern in this study (57). Descriptive statistics for all variables are presented in Table 9.

**Table 9:** Descriptive statistics of variables.

Variable and sub-dimensions	Min	Max	*M*	*SD*
Conflicting health information				
Objective conflict experience	1.00	5.00	3.64	0.72
Subjective conflict perception	1.00	5.00	3.61	0.85
**Disease uncertainty**	1.00	5.00	3.34	0.82
**Fear of disease progression**	1.00	5.00	3.04	0.91
Emotional response	1.00	5.00	3.22	0.90
Physiological health	1.00	5.00	3.14	0.94
Family and social function	1.00	5.00	2.43	1.26
**Tolerance of uncertainty**	1.00	5.00	2.89	0.97

#### 3.2.2 Correlation analysis

Pearson correlation analysis revealed significant positive associations among all variables: objective conflict experience, subjective conflict perception, Disease Uncertainty, fear of disease progression, and tolerance of uncertainty. The correlation matrix (Table 10) indicates that these relationships support further analysis of the proposed model.

**Table 10:** Results of correlation analysis.

Variable	1	2	3	4	5
1. Objective conflict experience	1				
2. Subjective conflict perception	0.678**	1			
3. Disease uncertainty	0.685**	0.598**	1		
4. Tolerance of uncertainty	0.361**	0.311**	316**	1	
5. Fear of disease progression	0.554**	0.485**	0.555**	0.591**	1

#### 3.2.3 Moderated mediation analysis

Moderated mediation analyses were conducted for both objective conflict experience (OCE) and subjective conflict perception (SCP) models. This study used the subscales of objective conflict experience and subjective conflict perception rather than a total score to provide a more nuanced understanding of how different aspects of conflicting health information perception relate to Disease Uncertainty and fear of progression. This approach aligns with Zimbres et al. (12) findings that objective exposure to conflicting information and subjective perceptions of such conflicts may yield distinct outcomes.

Results indicated relationships consistent with the proposed hypotheses (Tables 11, 12). The analysis found that both OCE and SCP showed positive associations with fear of disease progression (*β*_OCE_ = 0.18, *β*_SCP_ = 0.16, *p*s < 0.001), consistent with Hypothesis 1.

**Table 11:** Regression coefficients and model fit indices for moderated mediation analysis (OCE).

Regression equation		Overall model fit indices			Significance of regression coefficients			
Outcome variable	Predictor variable	*R*	*R^2^*	*F*	*β*	95% CI Lower Bound	95% CI Upper Bound	*t*
Disease uncertainty	Objective conflict experience	0.69	0.47	152.26^***^	0.65	0.58	0.72	18.55^***^
	Tolerance of uncertainty				0.08	0.02	0.15	2.40*
Fear of progression	Objective conflict experience	0.74	0.55	154.74^***^	0.18	0.11	0.27	4.48^**^
	Disease uncertainty				0.27	0.18	0.35	6.53^**^
	Tolerance of uncertainty				0.44	0.37	0.50	13.55^***^
	Disease uncertainty* Tolerance of uncertainty				0.14	0.10	0.19	6.07^**^

**Table 12:** Regression coefficients and model fit indices for moderated mediation analysis (SCP).

Regression equation		Overall model fit indices			Significance of regression coefficients			
Outcome variable	Predictor variable	*R*	*R2*	*F*	*β*	95% CI Lower Bound	95% CI Upper Bound	*t*
Disease uncertainty	Subjective conflict perception	0.61	0.37	101.49^***^	0.55	0.47	0.62	14.83^***^
	tolerance of uncertainty				0.15	0.07	0.22	3.99*
Fear of progression	Subjective conflict perception	0.74	0.55	152.93^***^	0.16	0.08	0.23	4.10^**^
	disease uncertainty				0.30	0.22	0.38	7.93**
	Tolerance of uncertainty				0.44	0.38	0.51	13.95^***^
	disease uncertainty* Tolerance of uncertainty				0.15	0.10	0.20	6.45^**^

Regarding the proposed mediation effect (Hypothesis 2), analysis revealed that both OCE and SCP were positively associated with disease uncertainty (*β*_OCE_ = 0.65, *β*_SCP_ = 0.55, *p*s < 0.001). Further, disease uncertainty showed positive associations with fear of disease progression in both models (*β*_OCE_ = 0.27, *β*_SCP_ = 0.30, *p*s < 0.001).

**Figure 4:**
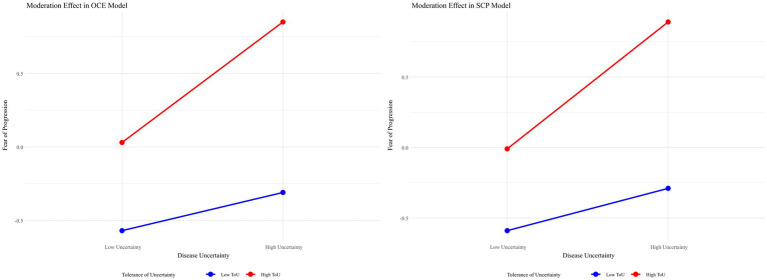
Moderating effect of tolerance of uncertainty on the relationship between disease uncertainty and fear of disease progression.

Concerning the proposed moderation effect (Hypothesis 3), tolerance of uncertainty significantly moderated the relationship between disease uncertainty and fear of disease progression in both models (*β*_OCE_ = 0.14, *β*_SCP_ = 0.15, *p*s < 0.001). Simple slope analyses revealed that for individuals with low tolerance of uncertainty, the effect of disease uncertainty on fear of disease progression was stronger (*β*_OCE_ = 0.41, *t* = 8.75; *β*_SCP_ = 0.45, *t* = 10.52; *p*s < 0.001), while for those with high tolerance of uncertainty, this effect was weaker (*β*_OCE_ = 0.12, *t* = 2.70; *β*_SCP_ = 0.15, *t* = 3.31; *p*s < 0.001), as illustrated in Figure 4.

These findings indicate that disease uncertainty partially mediates the relationship between conflicting health information (both objective and subjective) and fear of disease progression, with tolerance of uncertainty moderating the second stage of this mediation process. As individuals’ tolerance of uncertainty increases, the impact of disease uncertainty on fear of disease progression decreases (Table 13).

**Table 13:** Indirect effects at different levels of tolerance of uncertainty.

Effect type	Level	OCE model			SCP model		
		Effect	*SE*	95% CI	Effect	*SE*	95% CI
Indirect effect	*M* − 1 *SD*	0.08	0.03	[0.02, 0.14]	0.08	0.03	[0.02, 0.13]
	*M*	0.17	0.03	[0.11, 0.24]	0.17	0.03	[0.11, 0.22]
	*M* + 1 *SD*	0.29	0.04	[0.21, 0.37]	0.27	0.04	[0.21, 0.35]

## 4 Discussion

### 4.1 Major findings

This study, grounded in illness uncertainty theory, elucidates the relationship between conflicting health information and fear of disease progression among HPV-positive individuals, as well as the underlying mechanisms of this relationship. The findings reveal two key aspects of this dynamic. First, the study demonstrates how conflicting health information operates: it directly influences fear of disease progression in HPV-positive individuals while also exerting an indirect effect through the mediating role of disease uncertainty. Second, the research identifies when this effect is most pronounced: the latter part of the mediation process is moderated by tolerance of uncertainty. Specifically, among HPV-positive women, higher tolerance of uncertainty attenuates the impact of disease uncertainty on fear of disease progression. These results offer significant theoretical insights and practical implications for interventions aimed at addressing fear of disease progression in HPV-positive women.

In previous studies, no scale has been specifically designed to measure conflicting health information in the HPV context. This study developed and validated the HPV-Specific Conflicting Health Information Scale, drawing primarily from the frequently asked questions and concerns expressed by patients during HPV diagnosis and treatment. The scale development referenced Carpenter et al. (39) method for measuring conflicting medication information. Through in-depth interviews with HPV-positive individuals and rigorous expert consultations, an initial scale comprising 8 items was developed. Following validation with 218 participants and a test–retest reliability check with 42 participants, the scale demonstrated sound psychometric properties that meet the requirements for psychological measurement. The final scale effectively captures both objective experiences and subjective perceptions of HPV-related conflicting health information.

The results of this study demonstrate a positive correlation between conflicting health information and fear of disease progression among HPV-positive individuals. Both objective experiences of conflict and subjective conflict perceptions predicted fear of disease progression in HPV-positive women. This finding aligns with previous research suggesting that conflicting health information can lead to negative consequences. For instance, Nagler et al. (45) found that contradictory information about breast cancer screening led to confusion and anxiety among women.

Previous research on fear of disease progression has primarily focused on patients with diagnosed cancer or chronic diseases (46). This study extends this concept, confirming that fear of disease progression is also applicable to HPV-positive individuals. This finding emphasizes that even though not all HPV infections develop into cancer, HPV-positive individuals may experience fear of disease progression. It indicates that patients may face mental health challenges even in situations where disease prognosis is uncertain, providing a new perspective for understanding and intervening in HPV-related mental health issues.

Furthermore, this study validated the mediating role of disease uncertainty between conflicting health information and fear of disease progression. The results indicate that conflicting health information not only directly affects fear of disease progression but also indirectly influences it by increasing patients’ disease uncertainty. Specifically, when HPV-positive individuals encounter conflicting health information, they may experience uncertainty about their disease status. When the uncertainty is appraised as harmful, it would consequently lead to an increase in fear of disease progression. The identification of this mechanism enhances the understanding of the psychological state of HPV-positive individuals. It reveals how conflicting health information affects patients’ mental health by increasing uncertainty.

The study further investigated the moderating role of tolerance of uncertainty in the relationship between disease uncertainty and fear of disease progression. Results indicated that for individuals with higher tolerance of uncertainty, the impact of disease uncertainty on fear of disease progression was attenuated. This finding suggests that even when faced with the same degree of disease uncertainty (arising from identical conflicting health information), individuals may exhibit varying psychological responses. A potential explanation for this phenomenon is that individuals with high tolerance of uncertainty possess superior adaptive emotion regulation strategies and lower maladaptive emotion regulation tendencies (47). Consequently, they may be able to maintain a relatively calm state of mind when confronted with uncertain situations, rather than immediately succumbing to anxiety or panic.

### 4.2 Clinical implications

The findings of this study reveal the psychological landscape of HPV-positive individuals, highlighting the critical need for a comprehensive support strategy addressing fear of disease progression and conflicting health information.

For hospitals, several targeted interventions are recommended. First, doctor training should be strengthened to improve their awareness of patients’ psychological needs and communication skills. Second, hospitals can develop educational materials that specifically address common conflicting HPV-related health information with scientific explanations, helping patients better understand their condition and reduce uncertainty-related anxiety. Additionally, hospitals can facilitate support networks through both online and offline counseling services and organize patient communities for information and emotional exchange. Building on research evidence that demonstrates the malleability of uncertainty tolerance and its significant impact on mental health outcomes (48–50), hospitals can implement targeted psychological interventions. Studies have shown that brief interventions can effectively improve uncertainty tolerance and yield positive psychological outcomes (51), with Shapiro et al. (52) further emphasizing the potential for enhancing this capacity. These interventions may include exposure exercises, cognitive restructuring, and problem-solving training, as well as positive thinking-based approaches such as acceptance and commitment therapy, stress reduction, and emotion regulation techniques. Such comprehensive programs will help patients better manage uncertainty and cope with conflicting health information.

For physicians, it is essential to recognize and address conflicting health information during patient consultations. During diagnosis and treatment, physicians should provide clear, consistent information about what is normal, what requires intervention, and when follow-up is needed, thereby reducing uncertainty. They should also maintain good communication with patients, guiding patients to effectively utilize available hospital resources or online channels to alleviate psychological burden.

For patients, developing information screening skills and improving tolerance for uncertainty can help reduce psychological distress. In cases of severe distress, patients should be encouraged to seek professional help, participate in group counseling sessions, and learn effective coping strategies through these support systems.

### 4.3 Limitations and future directions

This study has several limitations that warrant careful consideration when interpreting the results. First, our expert consultation was limited both in number and geographic scope, primarily focusing on Wenzhou city. This restriction may hinder the applicability of findings to other regions or demographics. Second, the survey sample was confined to a single hospital, which could further limit generalizability across different settings or populations. Additionally, the participant sampling method was not comprehensive; we did not collect detailed demographic variables such as medical insurance status, economic conditions, and family social support. Lastly, the cross-sectional design hampers our ability to capture temporal dynamics within these psychological processes or establish definitive causal relationships.

Despite these limitations in sampling and methodology, our findings hold broader implications that merit further exploration on both theoretical and empirical fronts. One key area for future research is the need for more empirical evidence to elucidate universal aspects underlying psychological processes related to fear of disease progression while also recognizing context-specific variations. A recent meta-analysis by Sharpe et al. (53) highlighted this aspect by examining fear of disease progression in various chronic conditions such as diabetes and heart disease; it revealed consistent patient concerns about future impacts despite unique condition characteristics—often likened to a “Sword of Damocles” (53). This suggests that there may be fundamental psychological mechanisms at play across different illnesses.

To build upon these insights, subsequent studies should employ longitudinal designs to track how fear of progression evolves over time in diverse chronic conditions while considering individual patient demographics and healthcare experiences. Furthermore, exploring interventions aimed at alleviating these fears could provide valuable insights into effective coping strategies or communication techniques utilized by healthcare providers.

Another avenue for future research involves investigating additional buffering factors influencing fear of progression among HPV-infected individuals within cultural and organizational frameworks. The characteristics of healthcare systems—including their accessibility and affordability—can significantly shape how patients interpret conflicting health information amidst uncertainty surrounding their health status since access varies widely among individuals seeking professional guidance.

Moreover, prior studies indicate that contributors to fear of disease progression encompass both external signals from healthcare environments and internal emotional cues—a pattern evident across various cultures (31, 54, 55). However, while such relationships exist globally, they often differ in strength and expression depending on cultural contexts. For example, Asian philosophical traditions frequently emphasize embracing uncertainty as an intrinsic life element (56), potentially influencing how individuals cope with uncertainties related to health fears.

Future investigations should therefore consider conducting cross-cultural studies aimed at measuring how specific beliefs—shaped by sociocultural contexts—influence emotional responses toward health information management strategies regarding fear reduction concerning HPV progression outcomes. Qualitative interviews may also provide deeper insights into patients’ lived experiences with varying healthcare systems along with their perceptions regarding uncertainties linked with HPV-related health issues across diverse cultural settings.

## 5 Conclusion

This study demonstrates the significant impact of conflicting health information on fear of disease progression among HPV-positive individuals. Disease uncertainty plays a partial mediating role in the relationship between conflicting health information and fear of disease progression. Importantly, tolerance of uncertainty serves as a moderating factor, potentially buffering the negative effects of disease uncertainty on psychological outcomes. These findings highlight the psychological challenges faced by this population, even in the absence of cancer diagnosis, and underscore the need for clear, consistent health information dissemination to reduce uncertainty.

The results have important implications for public health strategies and clinical interventions. They suggest a two-pronged approach: reducing conflicting health information and enhancing individuals’ tolerance of uncertainty. Future research should focus on developing and testing interventions that provide accurate, consistent health information while simultaneously strengthening individuals’ capacity to cope with uncertainty. By addressing both the quality of health information and individuals’ psychological resources, public health initiatives can potentially mitigate the psychological burden associated with HPV infection and improve overall health outcomes.

## Data Availability

The raw data supporting the conclusions of this article will be made available by the authors without undue reservation.

## References

[1] CDC. (2024). Basic information about HPV and cancer. Cancer. Available at: https://www.cdc.gov/cancer/hpv/basic-information.html

[2] Wang W Arcà E Sinha A Hartl K Houwing N Kothari S. Cervical cancer screening guidelines and screening practices in 11 countries: a systematic literature review. Prev Med Rep. (2022) 28:101813. doi: 10.1016/j.pmedr.2022.101813, PMID: 35637896 PMC9142642

[3] Albright AE Allen RS. HPV misconceptions among college students: the role of health literacy. J Community Health. (2018) 43:1192–200. doi: 10.1007/s10900-018-0539-4, PMID: 29922992

[4] Singh D Vignat J Lorenzoni V Eslahi M Ginsburg O Lauby-Secretan B. Global estimates of incidence and mortality of cervical cancer in 2020: a baseline analysis of the WHO global cervical cancer elimination initiative. Lancet Glob Health. (2023) 11:e197–206. doi: 10.1016/S2214-109X(22)00501-0, PMID: 36528031 PMC9848409

[5] World Health Organization. (2024). Cervical cancer. Available at: https://www.who.int/news-room/fact-sheets/detail/cervical-cancer (Accessed November 6, 2024).

[6] World Health Organization. Global strategy to accelerate the elimination of cervical cancer as a public health problem. Geneva, Switzerland: World Health Organization (2020).

[7] Zhang J Zha T Wang X He W. Prevalence and genotype distribution of HPV infections among women in Chengdu, China. Virol J. (2024) 21:7–14. doi: 10.1186/s12985-024-02317-x, PMID: 38429823 PMC10908056

[8] Yilmaz E Eklund C Lagheden C Robertsson KD Lilja M Elfström M. First international proficiency study on human papillomavirus testing in cervical cancer screening. J Clin Virol. (2023) 167:105581. doi: 10.1016/j.jcv.2023.105581, PMID: 37688950

[9] Yang Y Liang L Meng J Liao X Cai R Ye H. Translation of the fear of progression questionnaire-parent version and its validation in parents of children with cancer. J Nurs Sci. (2022) 37:68–71. doi: 10.3870/j.issn.1001-4152.2022.01.068

[10] Perkins RB Guido RS Castle PE Chelmow D Einstein MH Garcia F. 2019 ASCCP Risk-Based Management Consensus Guidelines for Abnormal Cervical Cancer Screening Tests and Cancer Precursors. Journal of Lower Genital Tract Disease. (2020) 24:102–131. doi: 10.1097/LGT.0000000000000525 32243307 PMC7147428

[11] Carpenter DM Han PKJ. Conflicting health information In: Gulliver SB, Cohen LM, editors. Hoboken, NJ The Wiley encyclopedia of health psychology: John Wiley & Sons, Ltd. (2020). 47–53.

[12] Zimbres TM Bell RA Miller LMS Zhang J. When media health stories conflict: test of the contradictory health information processing (CHIP) model. J Health Commun. (2021) 26:460–72. doi: 10.1080/10810730.2021.1950239, PMID: 34304728

[13] Nagler RH Vogel RI Gollust SE Yzer MC Rothman AJ. Effects of prior exposure to conflicting health information on responses to subsequent unrelated health messages: results from a population-based longitudinal experiment. Ann Behav Med. (2022) 56:498–511. doi: 10.1093/abm/kaab069, PMID: 34398961 PMC9116588

[14] Barnwell PV Fedorenko EJ Contrada RJ. Healthy or not? The impact of conflicting health-related information on attentional resources. J Behav Med. (2022) 45:306–17. doi: 10.1007/s10865-021-00256-4, PMID: 34535867

[15] Carpenter DM Elstad EA Blalock SJ DeVellis RF. Conflicting medication information: prevalence, sources, and relationship to medication adherence. J Health Commun. (2014) 19:67–81. doi: 10.1080/10810730.2013.798380, PMID: 24015878 PMC8989251

[16] Santos B Blondon KS Sottas M Carpenter D Backes C Gessel EV. Perceptions of conflicting information about long-term medications: a qualitative in-depth interview study of patients with chronic diseases in the Swiss ambulatory care system. BMJ Open. (2023) 13:e070468. doi: 10.1136/bmjopen-2022-070468, PMID: 37940158 PMC10632873

[17] Ahn J Kahlor LA. When experts offer conflicting information: a study of perceived ambiguity, information insufficiency, trustworthiness and risk information behaviors. Health Commun. (2023) 38:3276–86. doi: 10.1080/10410236.2022.2146033, PMID: 36404712

[18] Wang L Gollust SE Rothman AJ Vogel RI Yzer MC Nagler RH. Effects of exposure to conflicting health information on topic-specific information sharing and seeking intentions. Health Commun. (2024):1–9. doi: 10.1080/10410236.2024.2350844, PMID: 38736132 PMC11554934

[19] Marshall LH Comello ML. Stymied by a wealth of health information: how viewing conflicting information online diminishes efficacy. J Commun Healthc. (2019) 12:4–12. doi: 10.1080/17538068.2019.1580064

[20] Hong SJ Kim Y. Relationship of exposure to contradictory information and information insufficiency to decision-making about HPV vaccination among south Korean college women. J Health Commun. (2023) 28:156–67. doi: 10.1080/10810730.2023.2191224, PMID: 36922760

[21] Gollust SE Fowler EF Nagler RH. Prevalence and potential consequences of exposure to conflicting information about mammography: results from nationally-representative survey of U.S. adults. Health Commun. (2023) 38:349–62. doi: 10.1080/10410236.2021.1951958, PMID: 34259097 PMC8758803

[22] Northouse LL. Mastectomy patients and the fear of cancer recurrence. Cancer Nurs. (1981) 4:213–20. doi: 10.1097/00002820-198106000-00004, PMID: 6909039

[23] Hinz A Mehnert A Ernst J Herschbach P Schulte T. Fear of progression in patients 6 months after cancer rehabilitation—a validation study of the fear of progression questionnaire FoP-Q-12. Support Care Cancer. (2015) 23:1579–87. doi: 10.1007/s00520-014-2516-5, PMID: 25412727

[24] Nagele E Reich O Greimel E Dorfer M Haas J Trutnovsky G. Sexual activity, psychosexual distress, and fear of progression in women with human papillomavirus-related premalignant genital lesions. J Sex Med. (2016) 13:253–9. doi: 10.1016/j.jsxm.2015.12.012, PMID: 26782607

[25] Rask M Oscarsson M Lindell G Swahnberg K. Women with abnormal pap smear result: a qualitative study of Swedish healthcare professionals’ experiences. Eur J Cancer Care. (2016) 25:980–91. doi: 10.1111/ecc.12415, PMID: 26545562

[26] Ciavattini A Delli Carpini G Giannella L Del Fabro A Banerji V Hall G. An online survey on emotions, impact on everyday life, and educational needs of women with HPV positivity or abnormal pap smear result. Medicine. (2021) 100:e27177. doi: 10.1097/MD.0000000000027177, PMID: 34766557 PMC8589238

[27] Li AS Luo CF Li AX Li F Wang LS Zhou MY. Development of HPV positive female disease progression fear scale and reliability and validity test. J Anhui Normal Univ (Nat Sci). (2021) 44:259–63. doi: 10.14182/J.cnki.1001-2443.2021.03.009

[28] Han PKJ Williams AE Haskins A Gutheil C Lucas FL Klein WMP. Individual differences in aversion to ambiguity regarding medical tests and treatments: association with Cancer screening cognitions. Cancer Epidemiol Biomarkers Prev. (2014) 23:2916–23. doi: 10.1158/1055-9965.EPI-14-0872, PMID: 25258015 PMC4257853

[29] McBride AA. Human papillomaviruses: diversity, infection and host interactions. Nat Rev Microbiol. (2022) 20:95–108. doi: 10.1038/s41579-021-00617-5, PMID: 34522050

[30] Mishel MH. Uncertainty in illness. Image: the. J Nurs Scholarsh. (1988) 20:225–32. doi: 10.1111/j.1547-5069.1988.tb00082.x 3203947

[31] Lee-Jones C Humphris G Dixon R Bebbington Hatcher M. Fear of cancer recurrence—a literature review and proposed cognitive formulation to explain exacerbation of recurrence fears. Psycho-Oncology. (1997) 6:95–105. doi: 10.1002/(SICI)1099-1611(199706)6:2<95::AID-PON250>3.0.CO;2-B, PMID: 9205967

[32] Boelen PA Reijntjes A. Intolerance of uncertainty and social anxiety. J Anxiety Disord. (2009) 23:130–5. doi: 10.1016/j.janxdis.2008.04.007 18565725

[33] Zvolensky MJ Vujanovic AA Bernstein A Leyro T. Distress tolerance: theory, measurement, and relations to psychopathology. Curr Dir Psychol Sci. (2010) 19:406–10. doi: 10.1177/0963721410388642, PMID: 33746374 PMC7978414

[34] Boelen PA Carleton RN. Intolerance of uncertainty, hypochondriacal concerns, obsessive-compulsive symptoms, and worry. J Nerv Ment Dis. (2012) 200:208–13. doi: 10.1097/NMD.0b013e318247cb17, PMID: 22373757

[35] Korkmaz H Güloğlu B. The role of uncertainty tolerance and meaning in life on depression and anxiety throughout Covid-19 pandemic. Personal Individ Differ. (2021) 179:110952. doi: 10.1016/j.paid.2021.110952, PMID: 34866725 PMC8631584

[36] Waroquier P Delevallez F Razavi D Merckaert I. Psychological factors associated with clinical fear of cancer recurrence in breast cancer patients in the early survivorship period. Psycho-Oncology. (2022) 31:1877–85. doi: 10.1002/pon.5976, PMID: 35674194

[37] Shen Z Zhang L Shi S Ruan C Dan L Li C. The relationship between uncertainty and fear of disease progression among newly diagnosed cancer patients: the mediating role of intolerance of uncertainty. BMC Psychiatry. (2024) 24:1–9. doi: 10.1186/s12888-024-06201-4, PMID: 39482640 PMC11526518

[38] Carpenter DM Geryk LL Chen AT Nagler RH Dieckmann NF Han PK. Conflicting health information: a critical research need. Health Expect. (2016) 19:1173–82. doi: 10.1111/hex.12438, PMID: 26709206 PMC5139056

[39] Carpenter DM DeVellis RF Fisher EB DeVellis BM Hogan SL Jordan JM. The effect of conflicting medication information and physician support on medication adherence for chronically ill patients. Patient Educ Couns. (2010) 81:169–76. doi: 10.1016/j.pec.2009.11.006, PMID: 20044230 PMC2891323

[40] Mishel MH. The measurement of uncertainty in illness. Nurs Res. (1981) 30:258–63. doi: 10.1097/00006199-198109000-00002, PMID: 6912987

[41] Ye Z She Y Liang M Knobf T Dixon J Hu Q. Revised Chinese version of Mishel uncertainty in illness scale: development, reliability and validity. Chinese Gen Pract. (2018) 21:1091–7.

[42] Carleton RN Norton MPJ Asmundson GJ. Fearing the unknown: a short version of the intolerance of uncertainty scale. J Anxiety Disord. (2007) 21:105–17. doi: 10.1016/j.janxdis.2006.03.014 16647833

[43] Zhang Y Song J Gao Y Wu S Song L Miao D. Reliability and validity of the intolerance of uncertainty scale-Short form in university students. Chin J Clin Psych. (2017) 25:285–8. doi: 10.16128/j.cnki.1005-3611.2017.02.020

[44] Hayes AF. Introduction to mediation, moderation, and conditional process analysis: a regression-based approach. New York, NY: Guilford Publications (2017).

[45] Nagler RH Yzer MC Rothman AJ. Effects of media exposure to conflict information about mammography: results from a population-based survey experiment. Ann Behav Med. (2019) 53:896–908. doi: 10.1093/abm/kay098, PMID: 30596830 PMC6735717

[46] Podina IR Todea D Fodor LA. Fear of cancer recurrence and mental health: a comprehensive meta-analysis. Psycho-Oncology. (2023) 32:1503–13. doi: 10.1002/pon.6205, PMID: 37596855

[47] Sahib A Chen J Cárdenas D Calear AL. Intolerance of uncertainty and emotion regulation: a meta-analytic and systematic review. Clin Psychol Rev. (2023) 101:102270. doi: 10.1016/j.cpr.2023.102270 36965452

[48] Benbassat J Pilpel D Schor R. Physicians’ attitudes toward litigation and defensive practice: development of a scale. Behav Med. (2001) 27:52–60. doi: 10.1080/08964280109595771, PMID: 11763825

[49] Fergus TA. Cyberchondria and intolerance of uncertainty: examining when individuals experience health anxiety in response to internet searches for medical information. Cyberpsychol Behav Soc Netw. (2013) 16:735–9. doi: 10.1089/cyber.2012.0671, PMID: 23992476

[50] Sarikaya O Civaner M Kalaca S. The anxieties of medical students related to clinical training. Int J Clin Pract. (2006) 60:1414–8. doi: 10.1111/j.1742-1241.2006.00869.x, PMID: 16787438

[51] Molton IR Koelmel E Curran M von Geldern G Ordway A Alschuler KN. Pilot intervention to promote tolerance for uncertainty in early multiple sclerosis. Rehabil Psychol. (2019) 64:339–50. doi: 10.1037/rep0000275, PMID: 31233326

[52] Shapiro MO Allan NP Raines AM Schmidt NB. A randomized control trial examining the initial efficacy of an intolerance of uncertainty focused psychoeducation intervention. J Psychopathol Behav Assess. (2023) 45:379–90. doi: 10.1007/s10862-022-10002-y

[53] Sharpe L Michalowski M Richmond B Menzies RE Shaw J. Fear of progression in chronic illnesses other than cancer: a systematic review and meta-analysis of a transdiagnostic construct. Health Psychol Rev. (2023) 17:301–20. doi: 10.1080/17437199.2022.2039744, PMID: 35132937

[54] Du L Cai J Zhou J Yu J Yang X Chen X. Current status and influencing factors of fear disease progression in Chinese primary brain tumor patients: a mixed methods study. Clin Neurol Neurosurg. (2024) 246:108574. doi: 10.1016/j.clineuro.2024.108574, PMID: 39357322

[55] Lim HL Suhail MK Lim CS Daher AM. Fear of progression, coping strategies, and associated factors among a sample of Malaysian women with breast cancer. Sci Rep. (2025) 15:922. doi: 10.1038/s41598-024-82143-x, PMID: 39762234 PMC11704191

[56] Shah M. Acceptance-based therapies and Asian philosophical traditions: similarities and differences in the concept of acceptance. J Ration Emot Cogn Behav Ther. (2021) 39:1–13. doi: 10.1007/s10942-020-00355-2

[57] Wen Z Tang D. Statistical approaches for testing common method bias: Problems and suggestions. Advances in Psychological Science. (2020) 28:215–223. doi: 10.16719/j.cnki.1671-6981.20200130, PMID: 30676241

